# Loss of function of *Colgalt1* disrupts collagen post-translational modification and causes musculoskeletal defects

**DOI:** 10.1242/dmm.037176

**Published:** 2019-06-17

**Authors:** Krista A. Geister, Alberto Jose Lopez-Jimenez, Scott Houghtaling, Tzu-Hua Ho, Roberto Vanacore, David R. Beier

**Affiliations:** 1 Center for Developmental Biology and Regenerative Medicine, Seattle Children's Research Institute, Seattle, WA 98105, USA; 2 Department of Pediatrics, Division of Genetics, University of Washington School of Medicine, Seattle, WA 98195, USA; 3 Center for Matrix Biology, Vanderbilt University Medical Center, Nashville, TN 37232, USA; 4 Department of Medicine, Division of Nephrology, Vanderbilt University Medical Center, Nashville, TN 37232, USA

**Keywords:** ENU, Collagen glycosylation, Collagenopathies, Myopathy, GLT25D1

## Abstract

In a screen for organogenesis defects in N-ethyl-N-nitrosourea (ENU)-induced mutant mice, we discovered a line carrying a mutation in *Colgalt1* [collagen beta(1-O)galactosyltransferase type 1], which is required for proper galactosylation of hydroxylysine residues in a number of collagens. *Colgalt1* mutant embryos have not been previously characterized; here, we show that they exhibit skeletal and muscular defects. Analysis of mutant-derived embryonic fibroblasts reveals that COLGALT1 acts on collagen IV and VI, and, while collagen VI appears stable and its secretion is not affected, collagen IV accumulates inside of cells and within the extracellular matrix, possibly due to instability and increased degradation. We also generated mutant zebrafish that do not express the duplicated orthologs of mammalian *Colgalt1*. The double-homozygote mutants have muscle defects; they are viable through the larvae stage but do not survive to 10 days post-fertilization. We hypothesize that the *Colgalt1* mutant could serve as a model of a human connective tissue disorder and/or congenital muscular dystrophy or myopathy.

## Abstract (precis)

**Summary:** The authors characterized a novel mouse mutant that has a defect in collagen glycosylation, which appears to affect muscle development. There is very little functional characterization of the affected gene, but this study provides analysis of its embryonic phenotype and the biochemistry of the null mutant, as well as the phenotype of null-mutant zebrafish.

## INTRODUCTION

There are nearly 30 different forms of collagen in vertebrates encoded by over 40 genes. This diverse array of proteins is responsible for maintaining the structural integrity of tissues. The vast majority of collagens are secreted by resident fibroblasts. Collagens are ultimately organized into a variety of superstructures, including fibrils, interrupted fibrils, beaded microfilaments, mesh networks and hexagonal lattices, and can even span the cell membrane. Many of them have been shown to interact with a number of additional extracellular matrix (ECM) components (Mienaltowski and Birk, 2014; Ricard-Blum, 2011).

The requirement of collagen in bone, cartilage, skin, muscle, ligaments and tendons is evident when one considers the phenotypes that result from their loss of function. There are a myriad of genetic causes assigned to what are now known as the ‘collagenopathies’, with many of them leading to severe disease phenotypes or lethality. Unfortunately, there are very few therapies available for these patients (Ricard-Blum, 2011; Jobling et al., 2014).

The volume of structural, genetic and functional diversity within this protein group is impressive (Mienaltowski and Birk, 2014; Ricard-Blum, 2011). However, they all begin their transit to the ECM in the same way as all secreted proteins – through the endoplasmic reticulum (ER). When three collagen subunits, or α-chains, from the same collagen type come into close proximity, they form a triple-helical trimer that will transit through the secretory pathway to the ECM (Mienaltowski and Birk, 2014; Ricard-Blum, 2011; Yamauchi and Sricholpech, 2012).

Collagens, and several collagen-like proteins, are unique in that their glycosylation takes place in the ER as opposed to the Golgi. As the α-chains are translated from their respective mRNAs into the ER, they are acted upon by a group of unique enzymes responsible for their post-translational modifications. These include proylyl and lysyl hydroxylases, which hydroxylate proline and lysine residues, and glucosyl- and galactosyltransferases, which add galactose to hydroxylysine or glucose to galactosylhydroxylysine, respectively (Yamauchi and Sricholpech, 2012; Kivirikko and Prockop, 1967).

One member of this collagen glycosylation cascade is COLGALT1, or collagen β(1-O)galactosyltransferase type I (Yamauchi and Sricholpech, 2012; Schegg et al., 2009), also called GLT25D1. *In vitro* studies have confirmed that COLGALT1 can galactosylate hydroxylysines in collagens I-V (Schegg et al., 2009), but its function *in vivo* remains to be elucidated. In fact, very little is known about the contribution of collagen glycosylation to collagen function, but it is hypothesized to lend stability to the trimer and the ultimate macromolecular structure (Yamauchi and Sricholpech, 2012).

We identified a mutant phenotype in a forward genetic screen for recessive developmental phenotypes that we named *fosse*. Whole-genome sequencing allowed us to perform homozygosity mapping, which indicated that the causal variant was in a region on mouse chromosome 8 (Chr8: 61958094-105272762) (Geister et al., 2017). Out of the seven variants we identified in this region, the variant in *Glt25d1*/*Colgalt1* seemed to be the most plausible candidate. Genotyping of additional affected embryos for the candidate variants we identified confirmed that the *fosse* phenotype is associated with the missense mutation in *Colgalt1*, which encodes collagen β(1-O)galactosyltransferase type I (Geister et al., 2017).

*Colgalt1^fosse/fosse^* embryos exhibit a number of defects, including perinatal lethality and a disorganization of muscle fibers. We describe here our characterization of this mutant phenotype. We demonstrate that the missense mutation in *Colgalt1* leads to a loss of COLGALT1 expression. We also provide evidence that COLGALT1 is required for proper glycosylation of collagens IV and VI, and that loss of its function reduces secretion of collagen I.

## RESULTS

### The *fosse* mutant phenotype is caused by a loss-of-function allele of *Colgalt1*

**Fig. 1.**
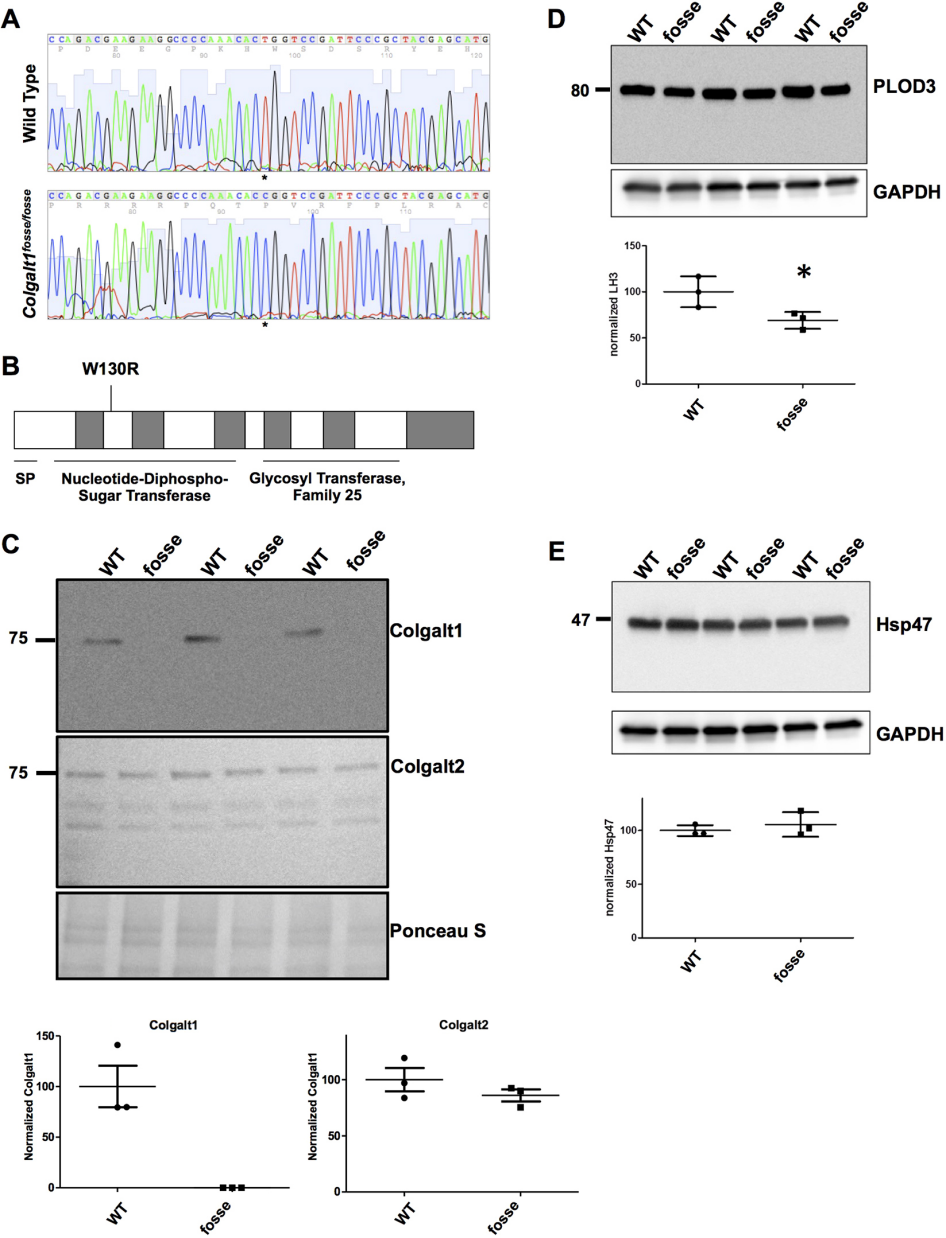
**Missense mutation in *Colgalt1* leads to loss of expression at the protein level.** (A) Chromatograms of wild-type and homozygous *fosse* sequence clearly demonstrates the substitution of a cytosine for a thymine (asterisks). (B) Schematic of COLGALT1 indicates where the missense mutation occurs in the encoded enzyme. (C) Immunodetection of COLGALT1 (top) and COLGALT2 (middle) by western blotting in wild-type and *fosse* MEF lysates. Ponceau S staining of the proteins transferred to the membrane (bottom) was used as loading control. Underneath these immunoblots is the quantitation by densitometric analyses of Colgalt1 and Colgalt2 chemiluminiscent signals. Molecular mass standard protein is shown in kDa for reference. (D,E) Immunoblot detection of PLOD3 (D) and Hsp47 (E) in wild-type and *fosse* MEF lysates. Signal intensity for bands of interest (*n*=3 in each group) was quantified with ImageJ and normalized to GAPDH (bottom). Densitometric quantitation for PLOD3 (D) and HSP47 (E) are shown underneath immunoblots. A statistically significant difference (*P*≤0.05) is indicated by an asterisk (*).

The *Colgalt1^fosse^* mutation substitutes an arginine for a highly conserved tryptophan in the nucleotide-diphospho-sugar transferase domain of COLGALT1 (c.T388C:p.W130R), which is in the N-terminal domain of the enzyme (Fig. 1A,B). The Polyphen score for this substitution is 1.0 (highly damaging) (Adzhubei et al., 2010). Western blot analysis of primary mouse embryonic fibroblast (MEF) lysates indicates that this mutation results in a loss of COLGALT1 protein expression (Fig. 1C). In all three independent mutant MEF lines, there is no detectable band corresponding to COLGALT1, which is expressed in all wild-type MEF lines (Fig. 1C, top panel). Western blots using primary antibodies against the paralog COLGALT2 (Fig. 1C, middle panel) and the related enzyme procollagen-lysine, 2-oxoglutarate 5-dioxygenase 3 (PLOD3) (Fig. 1D) showed that these enzymes are present, but not overexpressed upon COLGALT1 loss of function. Densitometric analysis showed that PLOD3 protein level is even slightly decreased. As shown in Fig. 1F, levels of the collagen-specific chaperone HSP47 was not changed. These results suggest that the loss of *Colgalt1* in mutant MEFs did not trigger a major compensatory response by enzymes involved in hydroxylysyl galactosylation or the folding and secretion of collagen molecules.

### *Colgalt1^fosse/fosse^* embryos exhibit musculoskeletal phenotypes

**Fig. 2.**
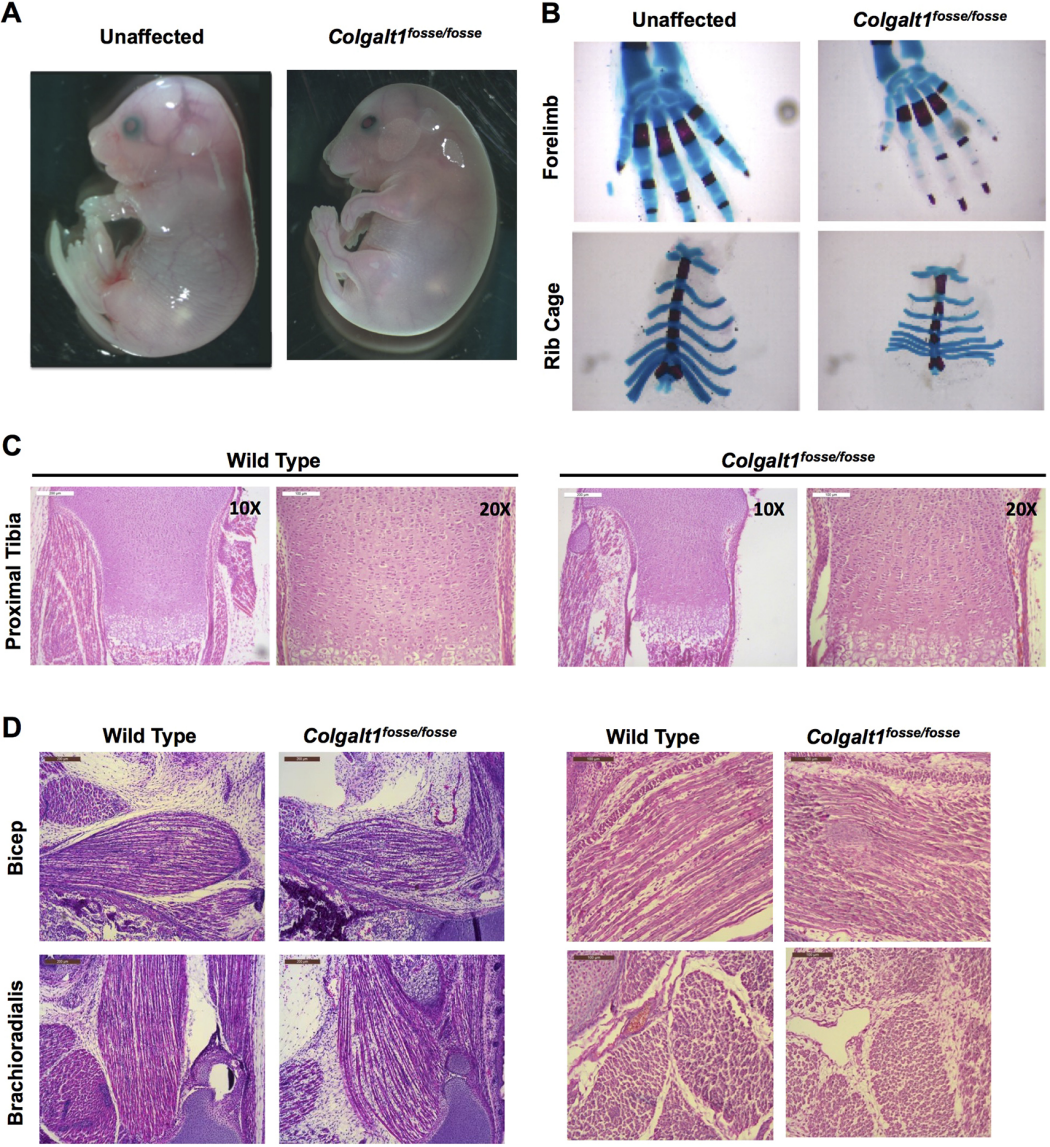
**Loss of function of *Colgalt1* leads to defects in musculoskeletal structure.** (A) *Colgalt1^fosse/fosse^* embryos are smaller, rounded and swollen compared to unaffected littermates at E18.5. (B) Skeletal preparations of *Colgalt1^fosse/fosse^* embryos at E18.5 clearly demonstrate a reduction in the size of the bones in the wrist and the rib cage. (C) Hematoxylin and Eosin (H&E)-stained sections through the proximal tibiae indicate that there is no gross disorganization in the growth plates of this skeletal element at E18.5. (D) H&E-stained sagittal sections through the forelimbs show disorganized, ragged and shorter muscle fibers in the bicep and brachioradialis compared to wild type. Right hand panels show magnified views of sections in left hand panels. Scale bars: 200 μm (C, 10×; D, left) and 100 μm (C 20×; D, right).

*Colgalt1^fosse/fosse^* embryos have a rounded body, appear slightly swollen and are smaller than unaffected littermates (Fig. 2A). The forepaws of mutant embryos are distinctly bent downward at the wrist (Fig. 2A). Staining of skeletal elements using Alcian Blue to label cartilage and Alizarin Red to stain mineralized tissue revealed that the carpals in the wrist and the rib cage are smaller than in wild-type mice (Fig. 2B). Hemotoxylin and Eosin (H&E) staining of histological sections through isolated tibiae revealed normal epiphyseal growth plate architecture (Fig. 2C). Staining of sections through the limbs revealed a readily observable muscle defect (Fig. 2D,E). The muscle fibers in major muscle groups of the arms and legs appear shorter, more disorganized and with increased interfiber space (Fig. 2D). Unsurprisingly, muscle groups also appear smaller in size overall (Fig. 2E).

### Collagen I accumulates in MEFs and is reduced in MEF ECM

**Fig. 3.**
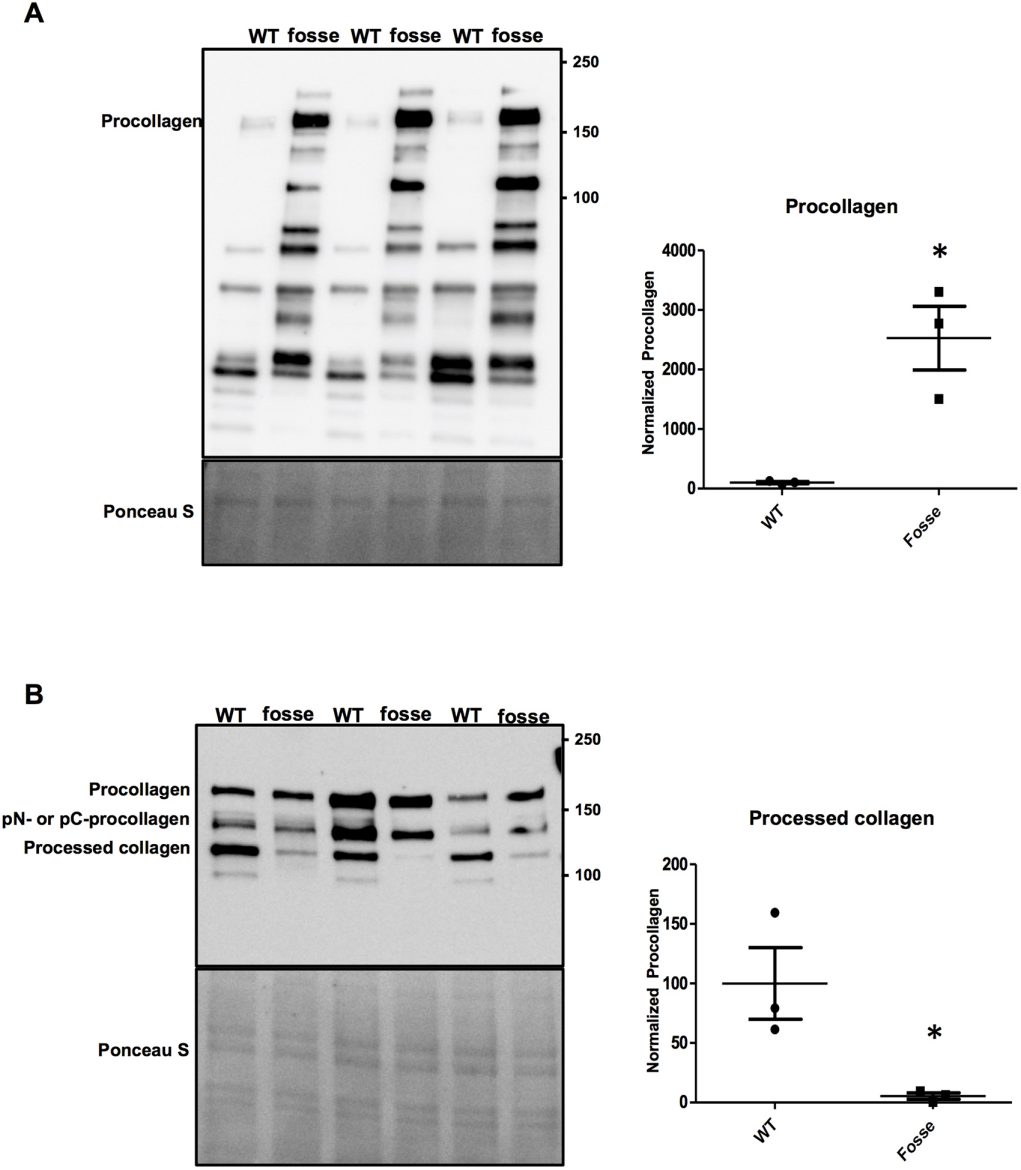
**The *Colgalt1^fosse^* mutation impairs secretion of collagen type I.** (A) Immunoblot detection of the α1 chain of type I collagen in wild-type and *fosse* MEF lysates. The electrophoretic migration of procollagen (i.e. full length, unprocessed form of the α1 chain of type I collagen) is indicated. Quantitation of the procollagen signal by densitometric analysis is shown adjacent to the immunoblot. (B) Immunoblotting analysis of type I collagen in culture medium of wild-type and *fosse* MEF lines. The migration of procollagen, amino (pN-) or carboxyl (pC-) procollagen proteolytic intermediates and processed collagen are indicated. Bands of interest were normalized to Ponceau S staining and quantified using ImageJ software (Schindelin et al., 2012). Quantitation of the processed collagen signal by densitometric analysis is shown adjacent to the immunoblot. The graphs show results from three independent experiments as mean (s.d.). Statistically significant difference was determined by unpaired *t-*test and it is indicated by an asterisk (*) above the data points (*n*=3 in each group; *P*≤0.05).

Collagen I is one of the most abundantly expressed and widely distributed collagen types (Mienaltowski and Birk, 2014; Ricard-Blum, 2011; Yamauchi and Sricholpech, 2012). To test whether type I collagen expression is altered in *Colgalt1^fosse/fosse^*, we conducted western blot analyses using a primary antibody against type I collagen (Fig. 3A,B).

Analysis of cell lysates would provide information about general expression and processing of type I collagen. The analysis of isolated ECM would determine whether there was impaired secretion or integrity, or a predisposition to degradation. Our western blot analyses clearly demonstrated that type I collagen accumulates intracellularly (Fig. 3A). Consistent with that observation, while its precursor (procollagen) and intermediate (pN- or pC-procollagen) forms are present, processed type I collagen is significantly decreased in the culture medium of mutant MEFs (Fig. 3B).

### Collagen IV modification and stability are disrupted in *Colgalt1^fosse/fosse^* MEFs, but expression in mutant tissue is normal

Collagen IV is one of the most highly glycosylated forms of collagen (Yamauchi and Sricholpech, 2012; Basak et al., 2016) and is crucial for maintenance of the basal lamina of epithelia (Poschl et al., 2004; Khoshnoodi et al., 2008; Brown et al., 2017). Mutations in collagen IV α-chain-encoding genes are known to cause vascular, eye and kidney defects (Pedchenko et al., 2010; Gould et al., 2006; Hudson et al., 1993). Additionally, it is one of the major forms of collagen disrupted upon loss of *Plod3* function in mutant mice (Rautavuoma et al., 2004; Sipila et al., 2007), which have a phenotype we found to be strikingly similar to that of *Colgalt1^fosse/fosse^* mutants (Geister et al., 2017). Taken together, these observations led to the hypothesis that collagen IV could be perturbed in *Colgalt1^fosse/fosse^* embryos.

**Fig. 4.**
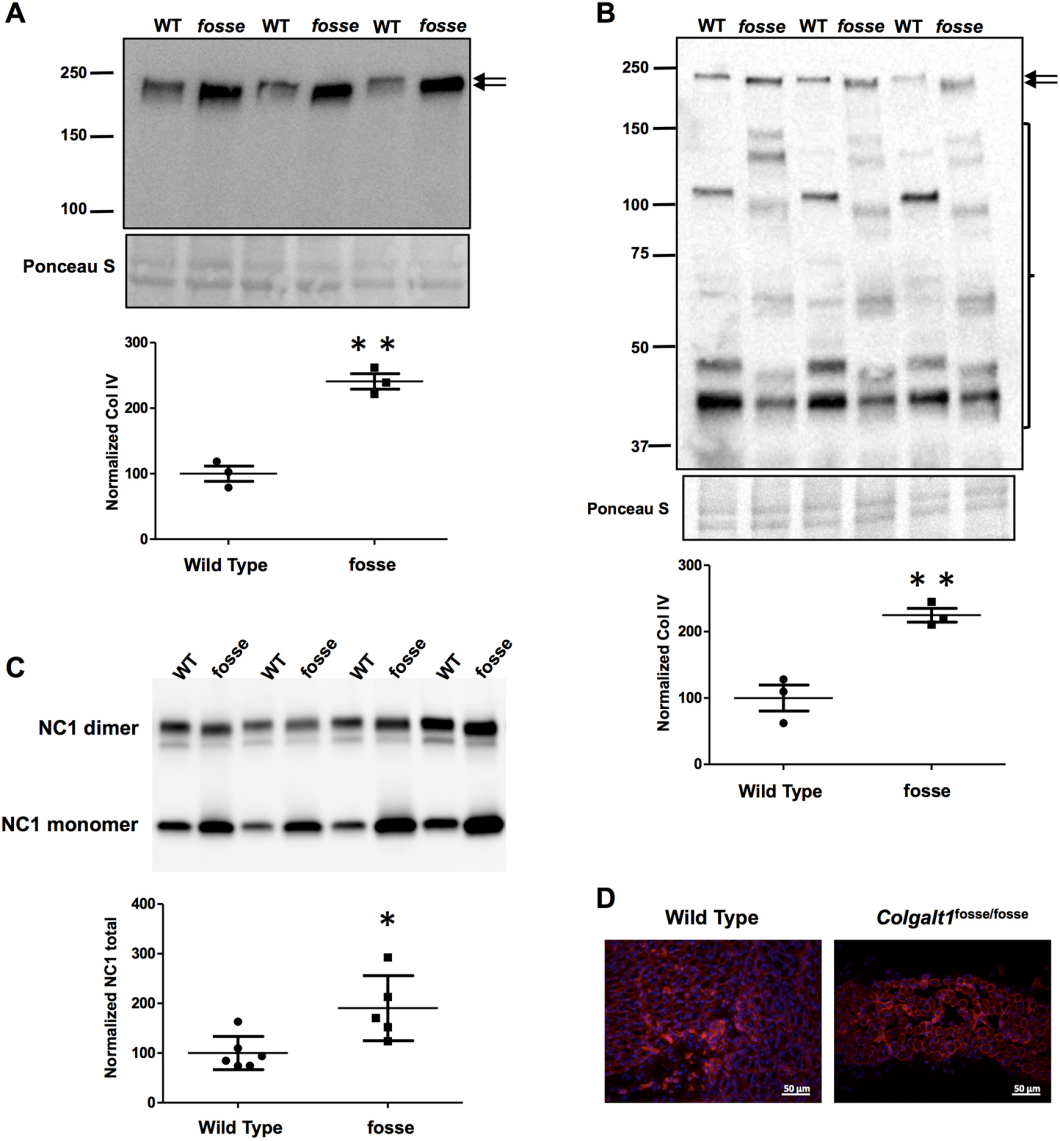
**Collagen IV accumulates intracellularly and exhibits decreased stability and molecular mass in *Colgalt1* mutant MEFs.** Immunoblot detection of the α2 chain of type IV collagen in lysates (A) and conditioned media (B) from wild-type and *Colgalt1^fosse/fosse^* MEF cultures. Ponceau S staining is shown as a loading control. Arrows indicate the band corresponding to full-length collagen IV. Graphs show densitometric quantitation of COL4A2 signal intensities normalized to Ponceau S. (C) Immunoblot detection of monomer and dimers of the NC1 domain of COL4A2 in collagenase digests of the ECM isolated from wild-type and *fosse* MEFs. The graph shows densitometric quantitation of the total (monomers and dimers) signal intensity from five different samples in each MEF group. Results are shown as mean (s.d.) and *P*-values ≤0.05 and ≤0.01 are indicated by one (*) or two (**) asterisks, respectively. (D) Immunofluorescent detection of type IV collagen in muscle tissue from wild-type and *fosse* mice. Magnification: 40×.

To test this hypothesis, we conducted western blots on MEF cultures to identify any anomalies in collagen IV processing, secretion or stability (Fig. 4A,B). Collagen IV accumulates in mutant MEFs (Fig. 4A), and the protein itself runs at a lower molecular mass (Fig. 4A,B). This shift is likely due to the loss of COLGALT1-mediated galactosylation of hydroxylysines. In the medium, the band corresponding to full-length collagen IV is more robust in mutant cells (Fig. 4B, arrows). Consistent with the increased secretion of collagen IV, an independent assay showed its increased deposition as evidenced by increased levels of non-collagenous (NC1) domain solubilized from the ECM produced by *fosse* MEFs (Fig. 4C,D). However, the collagen IV that is present in the mutant cell lines' media exhibits a banding pattern that is characteristic of protein degradation (Fig. 4B, bracket). These results suggest that *fosse* MEF cultures increase collagen IV production and deposition likely in response to its decreased stability due to defective lysine glycosylation.

Given that collagen IV is expressed in muscle and we have a muscle defect in our mutant mouse embryos, we decided to test expression of collagen IV by immunohistochemistry (IHC) in sections through *Colgalt1^fosse/fosse^* limbs. We used the same primary antibody used for western blots, which recognizes the NC1 domain of COL4A2 (Fig. 4E). Collagen IV should be expressed around individual muscle fibers and the group itself. Our IHC images clearly demonstrate that, in spite of the tissue organization differences, the expression pattern of COL4A2 in *Colgalt1^fosse/fosse^* muscle is similar to wild type. However, the COL4A2 that is present may not be as stable (Fig. 4B).

### Collagen VI is not properly glycosylated in *Colgalt1^fosse/fosse^* MEFs, but is expressed normally in mutant skin and muscle

Like collagen IV, collagen VI is another highly glycosylated form of collagen that is also expressed around individual muscle fibers (Cescon et al., 2015). Furthermore, mutations in collagen VI α-chain-encoding genes are a known cause of muscle disorders in humans (Bushby et al., 2014; Bernardi and Bonaldo, 2013). We hypothesized that loss of COLGALT1 function could lead to aberrations in collagen VI processing or secretion.

**Fig. 5.**
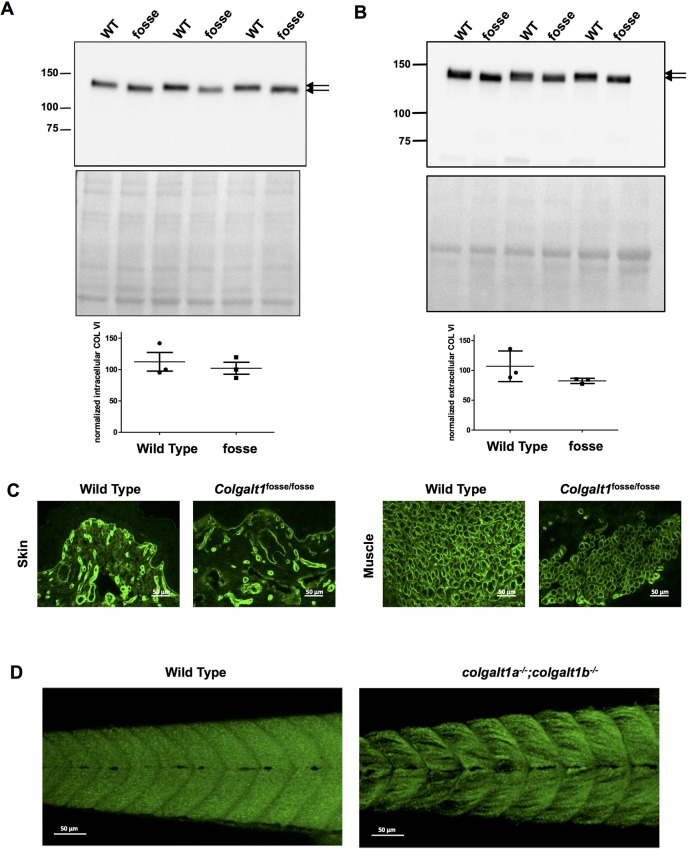
**Molecular mass of collagen VI is reduced upon loss of *Colgalt1* function in MEFs.** Immunoblot detection of the α1 chain of type VI collagen in lysates (A) and conditioned media (B) from wild-type and *fosse* MEF cultures. The double arrows indicate the difference in electrophoretic mobilities between the collagen VI molecule derived from wild-type and *fosse* MEF. Immunoblots were subjected to densitometric analyses with ImageJ using Ponceau S staining for normalization. (C) Immunofluorescent detection of type VI collagen in muscle tissue from wild-type and *fosse* mice. Magnification: 40×. (D) Phalloidin staining of wild-type and double-homozygous *colgalt1* mutant zebrafish at day 9 post-fertilization reveals muscle disorganization.

Using a primary antibody against COL6A1, we conducted western blot analyses on MEF lysates and cell culture medium. In both cases, we observed a reduction in molecular mass in mutant samples (Fig. 5A,B). As with collagen IV, this likely indicates the loss of galactosylation of hydroxylysines in COL6A1 upon loss of function of *Colgalt1*. Secretion of COL6A1 appears to be normal, as the bands are of the same intensity as in wild-type cell lysates and medium (Fig. 5A,B). The presence of a single band with minimal variation in molecular mass and lack of degradation fragments suggest that, unlike collagen IV, defective glycosylation does not significantly impact the stability of collagen VI.

Given that we have an observable defect in the musculature of our mutant embryos, and the fact that collagen VI does appear to be of a lower molecular mass in these mutants, we decided to test whether expression of COL6A1 was altered in mutant tissues by conducting IHC. We applied a primary antibody against COL6A1 to frozen sections through *Colgalt1^fosse/fosse^* limbs and observed normal expression in skin and muscle (Fig. 5C).

### Mutation of *colgalt1* in zebrafish causes early lethality

To complement our work in *Colgalt1^fosse/fosse^* mouse embryos, we generated *colgalt1* mutant lines of zebrafish using CRISPR-Cas9 genome-editing techniques (Varshney et al., 2015; Varshney et al., 2016). Like many genes in the zebrafish, *colgalt1* is duplicated, with loci corresponding to the *colgalt1* sequence on chromosomes 1 and 3. Hereafter, these loci are referred to as *colgalt1b* and *colgalt1a*, respectively. We selected single-guide RNAs (sgRNAs) that target the fifth exons of both genes (Varshney et al., 2016) and injected them into zebrafish embryos at the one-cell stage. This led to the generation of a 1 bp deletion in *colgalt1b* on chromosome 1 (*colgalt1b^1bpdelex5^*) and an 11 bp deletion/19 bp insertion in *colgalt1a* on chromosome 3 (*colgalt1a^11bpdel19bpinex5^*) (Fig. S1). Predicted open reading frames for the encoded proteins indicate that both mutations cause frameshifts followed by premature stop codons, which should lead to loss of *colgalt1a* and *colgalt1b* activity in double-homozygous fish (Fig. S1).

Crosses of double-heterozygous *colgalt1a* and *colgalt1b* mutant fish revealed that no double homozygotes were obtained at 3 months of age (Table 1). Single *colgalt1a* or *colgalt1b* homozygous fish were obtained at expected numbers, with no evident developmental defects. To more specifically determine the day of death, intercrosses were done between fish that were homozygous for one *colgalt1* allele and heterozygous for the other; one-quarter of the progeny should be double homozygotes. These were found to be present at days 7-9 post-fertilization and absent by day 10 (Table 2). Assessment of double-homozygous larvae at 8 and 9 days post-fertilization by phalloidin staining revealed gross morphological defects in muscle fiber organization (Fig. 5D), and larvae demonstrated reduced mobility at these ages.

**Table 1.**
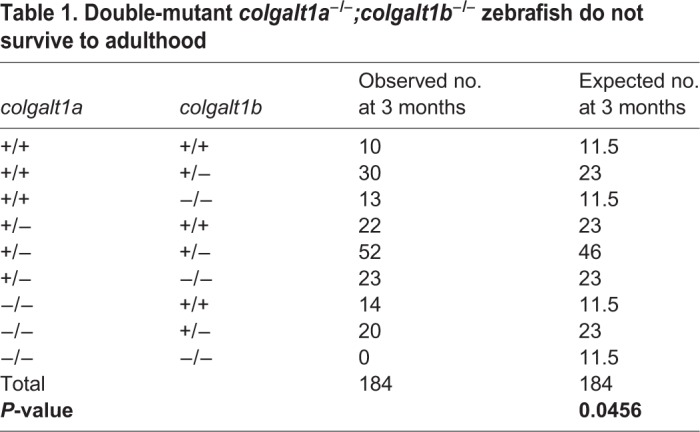
**Double-mutant *colgalt1a^−/−^;colgalt1b^−/−^* zebrafish do not survive to adulthood**

**Table 2.**
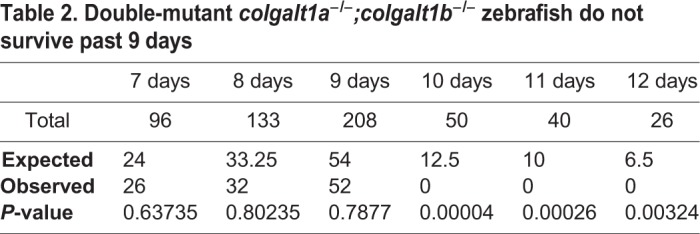
**Double-mutant *colgalt1a^−/−^;colgalt1b^−/−^* zebrafish do not survive past 9 days**

### The *Colgalt1^fosse/fosse^* skin barrier is intact

Reduction of type I collagen in the skin could disrupt the organ's ability to reduce fluid loss, leading to severe dehydration and death. To test the barrier function of *Colgalt1^fosse/fosse^* skin, we conducted a skin barrier assay (Indra and Leid, 2011). This test makes use of the intrinsic β-galactosidase activity of the skin at its basal layers. Fresh skin tissue is incubated with X-gal solution. Normal skin will keep the X-gal out of the basal layers of the skin, while a disrupted skin barrier will allow the X-gal to reach these basal layers, where β-galactosidase will catalyze the substrate to a blue by-product, turning the skin blue. Examination of homozygous mutant mice reveals that the skin barrier is intact (Fig. S2).

## DISCUSSION

We have previously reported the discovery of an N-ethyl-N-nitrosourea (ENU)-induced mutation of *Colgalt1* in the mouse (Geister et al., 2017) (Fig. 1A). Our characterization of its phenotype here provides valuable insight into the functions and tissue requirements for this enzyme in collagen processing. Clearly, COLGALT1 activity is required in muscle for proper architecture of muscle fibers within individual muscle groups (Fig. 2D,E). Previous work has also shown that COLGALT1 is capable of galactosylating hydroxylysine residues in collagens I-V *in vitro* (Schegg et al., 2009). Here, we have confirmed that COLGALT1 galactosylates hydroxylysine residues in collagen IV (Fig. 4A,B), and provide the first evidence indicating that COLGALT1 acts on collagen VI (Fig. 5A,B).

The *Colgalt1^fosse^* mutation is a missense mutation that substitutes an arginine for a highly conserved tryptophan residue (p.W130R, Fig. 1) (Geister et al., 2017). Previous studies on the functional domains of human COLGALT1 have shown that the C-terminal domain is required for galactosyltransferase activity (Perrin-Tricaud et al., 2011) and that the N-terminal domain shares homology with other galactosyltransferases and PLOD3 (Liefhebber et al., 2010). Our analysis of COLGALT1 expression indicates that this mutation leads to a loss of COLGALT1 in mutant MEFs (Fig. 1C). As it is a missense mutation, this change in protein expression is not likely due to nonsense-mediated decay. It is possible that the substitution of a basic amino acid for a hydrophobic amino acid in this highly conserved region results in protein instability and degradation.

*Colgalt1^fosse^* mutant embryos exhibit many structural phenotypes (Fig. 2), including bent wrists. While smaller carpals (Fig. 2B) could contribute to the wrist phenotype, it could also be due to a defect in muscle or connective tissue. Since we have observed a muscle defect, this phenotype could be indicative of contractures (Fig. 2D,E). It is worth noting that removing the skin of the lower, but not upper, limbs frequently results in complete disarticulation of the tibia from the femur, suggesting a weakening of the connective tissues in the knee joint. Upon dissection, the integument also feels weakened and is frequently marked with areas of hemorrhaging. In addition, at least one, if not both, of the eyelids are open in most mutant embryos (Fig. 2A). Analysis of skin barrier function revealed this to be normal (Fig. S2).

Mutant embryos also variably express cleft palate and exencephaly (Fig. S2); these phenotypes are never observed in wild-type littermates. Chi-square analysis indicates that this association is highly significant, demonstrating that cleft palate and exencephaly are contingent on the presence of the *fosse* mutation (Geister et al., 2017). Genomic analyses followed by Sanger sequencing of candidate ENU variants have suggested that it is unlikely that additional genetic loci are acting in a recessive or dominant manner to cause cleft palate, or in a dominant manner to cause exencephaly (Geister et al., 2017). We have concluded that these phenotypes are likely due to stochastic events during embryonic development.

We also investigated the role of *Colgalt1* in the development of zebrafish. The *Danio rerio* genome is partially duplicated, and zebrafish carry two orthologs of *Colgalt1*. These appear to be functionally redundant, as zebrafish homozygous for null mutations in either of these alleles survive to adulthood, appear normal and are fertile. This is also true for zebrafish that are homozygous for one mutant allele and heterozygous for the other. However, zebrafish homozygous for both null mutant alleles demonstrate defects in muscle development and do not survive to 10 days post-fertilization, potentially due to reduced mobility and inability to feed.

This is the first characterization of the embryonic phenotype of a *Colgalt1* null allele in the mouse. A recent characterization of heterozygous phenotypes in a *Colgalt1* ‘knock out’ also reports that the homozygous mutants are lethal prior to embryonic day (E)13.5 (Ye et al., 2018). This difference is likely due to the ENU-induced missense mutant retaining a low amount of activity (despite our observation that the protein is not detectable by western blot analysis).

Of note, *Colgalt1* has the potential to functionally and phenotypically overlap with the lysyl hydroxylases *Plod1*, *Plod2* and *Plod3* (Yamauchi and Sricholpech, 2012)*.* As mentioned previously, COLGALT1 and PLOD3 colocalize in the ER and are thought to work together to hydroxylate and glycosylate lysine residues in collagens (Schegg et al., 2009; Liefhebber et al., 2010). Furthermore, while categorized as a lysyl hydroxylase, PLOD3 also exhibits collagen galactosyl- and glucosylgalactosyltransferase activity (Heikkinen et al., 2000; Rautavuoma et al., 2002; Wang et al., 2002a,b).

Intriguingly, in the screen in which we discovered *Colgalt1^fosse/fosse^*, we also discovered mutant mice with an ENU-induced missense mutation of a conserved valine residue in *Plod3* (*Plod3^ugli/ugli^*; c.T695A:pV232E) that phenocopies *Colgalt1^fosse/fosse^* (Geister et al., 2017). The *Plod3^ugli/ugli^* mutation occurs in exon 7, closer to the N-terminus and is only five residues downstream of a mutation reported in a *PLOD3* compound heterozygous human patient with multiple abnormalities (p.N223S; Salo et al., 2008). Below, we compare our two mutant phenotypes with those that have been previously described to illustrate how these new mutant alleles fit within the current model of post-translational modification of collagens.

*Colgalt1^fosse/fosse^* and *Plod3^ugli/ugli^* mutant embryos survive until late gestation (E18.5), and presumably die at or around the time of birth, as we have never observed a homozygous live pup from either line. This is in contrast to all of the *Plod3* mutant alleles that have been described, which die between E8.5 and E14.5 (Rautavuoma et al., 2004; Ruotsalainen et al., 2006). PLOD3-mediated glycosylation of collagen IV is critical for basement membrane formation and is required for survival through midgestation (Rautavuoma et al., 2004). The hypomorphic allele of *Plod3* dies by E14.5, whereas an engineered missense mutation that eradicates lysyl hydroxylase activity survives after birth (Ruotsalainen et al., 2006). Therefore, we have discovered two mutant alleles that allow enough glucosylgalactosyltransferase activity of PLOD3 and galactosyltransferase activity of COLGALT1 to ensure survival past midgestation, but ultimately insufficient levels to allow survival past birth. Given the nearly identical phenotypes of *Colgalt1^fosse/fosse^* and *Plod3^ugli/ugli^*, it is likely that both are acting in the same cell types and catalyzing hydroxylation and glycosylation of the same collagen α-chains.

Specifically, we present evidence that COLGALT1 acts on collagen IV and VI (Figs 4 and 5). Both of these types exhibit a reduction in molecular mass that is likely due to the loss of glycosylation of hydroxylysines (Figs 4A,B; 5A,B). Galactose is the first sugar to be added in the collagen glycosylation cascade (Spiro, 1967). Thus, loss of COLGALT1 function will also likely disrupt glucosylgalactosyltransferase activity. These alterations in glycosylation of collagen IV and VI do not affect their expression in tissues (Figs 4C; 5C). Collagen VI appears stable and its secretion is not affected (Fig. 5A,B). However, collagen IV accumulates inside of cells and within the ECM (Fig. 4A-C), possibly due to instability and increased degradation (Fig. 4B; Rautavuoma et al., 2004).

We propose that these defects in collagen processing and secretion account for the observed phenotypes in *Colgalt1^fosse/fosse^* embryos; specifically, disruptions of collagen IV and VI could cause muscle fiber disorganization (Sipila et al., 2007; Bushby et al., 2014; Kalluri, 2003). We also suggest that it is likely that mutations of *COLGALT1* would cause connective tissue disorders in humans, similar to the patient with a defect in PLOD3 expression whose phenotype included bone fragility and contractures (Salo et al., 2008). Of note, a recent report described compound heterozygous mutations of COLGALT1 in two pediatric patients with cerebral small vessel disease (Miyatake et al., 2018). COLGALT1 was investigated because of the known role of COL4A1/COL4A2 in similar diseases (Weng et al., 2012; Jeanne et al., 2012). Neither patient was reported to have primary muscle disease. Given the role of COLGALT1 in the metabolism of many collagens, including those such as COL6A1, which is associated with primary myopathies (Kirschner, 2013), we speculate that these patients retained sufficient low-level enzyme activity to spare them from the embryonic defects seen in our mutant and the related *Plod3* mutants.

Additional studies of *Colgalt1* and related enzymes could add clarification to the complex process of collagen post-translational modification. Although we know the cellular locale, key players and tissue requirements for collagen glycosylation, the benefit that these modifications provide remains elusive. Future studies of COLGALT1 and related enzymes will expand our understanding of collagen biosynthesis and its involvement in disorders of collagen glycosylation.

## MATERIALS AND METHODS

### Generation, care and use of animals

All animal work was reviewed and approved by the Seattle Children's Research Institute's Institutional Care and Use Committee (IACUC). The discovery and positional cloning of the *Colgalt1* mutant line was previously described (Geister et al., 2017). The line was generated and maintained on the C57BL/6J background. Genotype analysis was done by PCR with primers (F: 5′-GCCACCTCTGTGAAGTCCTC-3′, R: 5′-CCATTCTATCCTCCCTGTGG-3′) to generate a 265 bp product followed by digestion with *Msp*I. The ENU mutation introduces an *Msp*I site.

### Generation and culture of mouse embryonic fibroblasts

MEFs were derived from E14.5 embryos using standard procedures (Joyner, 2000; Nagy, 2003). MEFs were cultured at 37°C and 5% CO_2_ in DMEM supplemented with 10% fetal bovine serum (FBS), 1% penicillin-streptomycin and 50 µg/ml ascorbic acid. After reaching confluency, cells were cultured in FBS-free medium for an additional 40 h before collecting culture media and preparing whole-cell extracts. For analyses of collagen deposition in the ECM, MEFs were cultured for 7 days past confluency with daily changes of complete media.

### Western blotting protocols

For lysate preparation, MEFs were washed twice with PBS and lysed with RIPA buffer supplemented with HALT Protease Inhibitor Cocktail (Thermo Fisher Scientific). Culture medium was harvested and concentrated about ten times with 10K MWCO Amicon Ultra Centrifugal filters (Millipore). For the analyses of collagen IV present in the insoluble ECM, MEF cultures (7 days post-confluency) were suspended in 1% (w/v) deoxycholate and homogenized by sonication. After centrifugation at 20,000 ***g*** for 15 min, the insoluble pellet (ECM) was digested with 50 µg/ml of purified bacterial collagenase (Worthington, cat. # LS005273) overnight at 37°C to solubilize the NC1 domains (Lopez-Jiménez et al., 2017).

For immunoblotting analyses, intracellular and extracellular (soluble and insoluble) protein extracts were fractionated on 4-20% SDS-PAGE and electro transferred onto nitrocellulose membranes. Membranes were incubated overnight with the following antibodies and the indicated dilutions: anti-NC1 collagen IV (H22, Chondex; cat. # 7071, 1:1000), anti-collagen I (MD Biosciences; cat. # 203002; 1:1000), anti-collagen VI (Abcam; ab182744; 1:2000), anti-PLOD3 (Proteintech; 11027-1-AP; 1:1000), anti-Hsp47 (Proteintech; 10875-1-AP; 1:1000), anti-Glt25d1/Colgalt1 (Proteintech; 16768-1-AP; 1:1000), anti-Glt25d2/Colgalt2 (Proteintech; 25993-1-AP; 1:500) and anti-GAPDH (Ambion; 6C5; 1:4000). Immunoblots were incubated with horseradish peroxidase (HRP)-conjugated donkey anti-rabbit, anti-rat or anti-mouse as secondary antibodies (Jackson ImmunoResearch Laboratories) for 1 h at room temperature followed by detection of HRP activity using Pierce enhanced chemiluminescence (ECL) reagents (Thermo Fisher Scientific). ImageJ (Schindelin et al., 2012) and GraphPad Prism v5.0 (GraphPad Software, La Jolla) were used for quantification and determination of statistical significance (unpaired *t*-test) of immunoblot experiments, respectively.

### Skeletal preparations

Skeletal preparations were performed as previously described (Ovchinnikov, 2009).

### Histological analyses

Tissues were collected from embryos at E18.5 and fixed overnight in 4% paraformaldehyde. They were then either processed for paraffin or frozen sectioning. For paraffin-embedded tissue processing, they were incubated overnight in PBS, dehydrated in ethanol to 70% and embedded in paraffin. For frozen sections, fixed tissues were incubated in 30% sucrose until the tissue sank, and embedded in OCT freezing medium on dry ice. Sections were then cut and mounted on slides. Paraffin-embedded sections were stained with H&E, whereas frozen sections were used for immunohistochemistry, the protocols for which are detailed below.

For immunofluorescence studies, frozen tissue sections were allowed to equilibrate to room temperature, washed in 1× PBS for 5 min to dissolve the OCT, and incubated with blocking solution for 1 h (blocking solution: 1× PBS, 3% bovine serum albumin, 0.3% Triton X-100, 0.02% sodium azide, 3% goat serum). Slides were then incubated with primary antibodies against COL4A2 (1:1000, rat monoclonal H22 antibody, Chondex, cat. # 7071,) or COL6A1 (1:100, Abcam, #6588). The next day, slides were washed in PBS and incubated with secondary antibodies. For COL4A2, the slides were incubated with goat anti-rat Alexa-Fluor-647 from Life Technologies (cat. # A21247) diluted 1:1000 for 1 h at room temperature. For COL6A1, we used goat anti-rabbit Alexa-Fluor-488 from Life Technologies (#a11008), diluted 1:500 and incubated for 1 h at room temperature. After washing the slides, the tissues that reacted with anti-COL4A2 antibodies were incubated with Vectashield^®^ antifade mounting medium with DAPI (Vector Laboratories, cat. # H-1200), whereas those that reacted with anti-COL6A1 antibodies were incubated with Hoechst 33258 solution (Life Technologies Corp., cat. # H3569) diluted 1:50,000 in PBS for 5 min, rinsed in PBS and mounted with aqueous mounting medium.

### Skin barrier assay

Embryos were collected at E18.5 and the skin barrier assay was performed as previously described (Indra and Leid, 2011).

### CRISPR-Cas9 genome editing in zebrafish

Mutations in the sgRNA target site 5′-GGGCTGTTTTGCGGTGCCGA-3′ in exon 5 of the *colgalt1* isoform on chromosome 1 and target site 5′-GGGATGCTTTGCGGTTCCAA-3′ in exon 5 of the *colgalt1* ortholog on chromosome 3 were generated using CRISPR-Cas9. *Cas9* mRNA was prepared from plasmid MLM3613 using the mMESSAGE mMACHINE™ T7 Transcription Kit (Thermo Fisher Scientific/Invitrogen, #AM1344). The sgRNA was prepared using a cloning-free synthesis method (Varshney et al., 2015). The DNA template was generated by annealing oligos and extending using High Fidelity Phusion Polymerase (New England BioLabs, #M0530S). *In vitro* transcription was performed using HiScribe™ T7 Quick High Yield RNA Synthesis Kit (New England BioLabs, #E2050S). Plasmid MLM3613 was a gift from Keith Joung (Addgene plasmid # 42251) (Hwang et al., 2013). A total of 2 μl of solution containing *Cas9* mRNA at 333 ng/µl and sgRNA at 24 ng/µl was injected into the yolk sac of single-celled zebrafish embryos. Screening of F1 progeny from injected embryos and genotyping of F2 progeny from double-heterozygous incrosses was performed by PCR and sequencing with F: 5′-GTCACACGATCTGTGCGTCT-3′, R: 5′-TGAACTTTATCTTTGCGTTTGACT-3′ for the isoform on chromosome 1 and F: 5′-ACTCAAACTTCTGGTGCGGA-3′, R: 5′-ATTGTGGCACACACCTGCTAT-3′ for the isoform on chromosome 3.

### Phalloidin staining

Phalloidin assay was performed as previously described (Goody et al., 2012).

## Supplementary Material

Supplementary information
