# Supplementary material for: Loss of function of Colgalt1 disrupts collagen post-translational modification and causes musculoskeletal defects
Source: Dis Model Mech. 2019 Jun 17;12(6):dmm037176. doi: 10.1242/dmm.037176 (PMC6602307; doi:10.1242/dmm.037176)
Supplement: Supplementary information [file dmm-12-037176-s1.pdf]

A

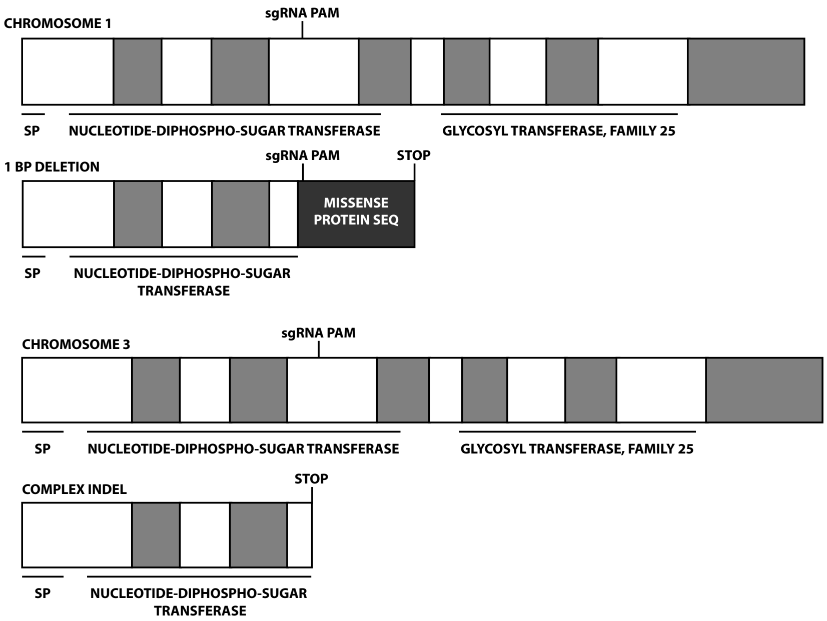

Figure S1.

A) In zebrafish *colgalt1* is duplicated, with loci on chromosomes 1 and 3. Targeting these at the sgRNA PAM sites shown led to the generation of a 1 bp deletion in *colgalt1b* on chromosome 1 (*colgalt1b*^{1bpdelex5}), and an 11 bp deletion/19 bp insertion in *colgalt1a* on chromosome 3 (*colgalt1a*^{11bpdel19bpinex5}). Predicted open reading frames for the encoded proteins indicate that both mutations cause frameshifts followed by premature stop codons, which should lead to loss of *colgalt1a* and *colgalt1b* activity in double homozygous fish.

Disease Models & Mechanisms • Supplementary information

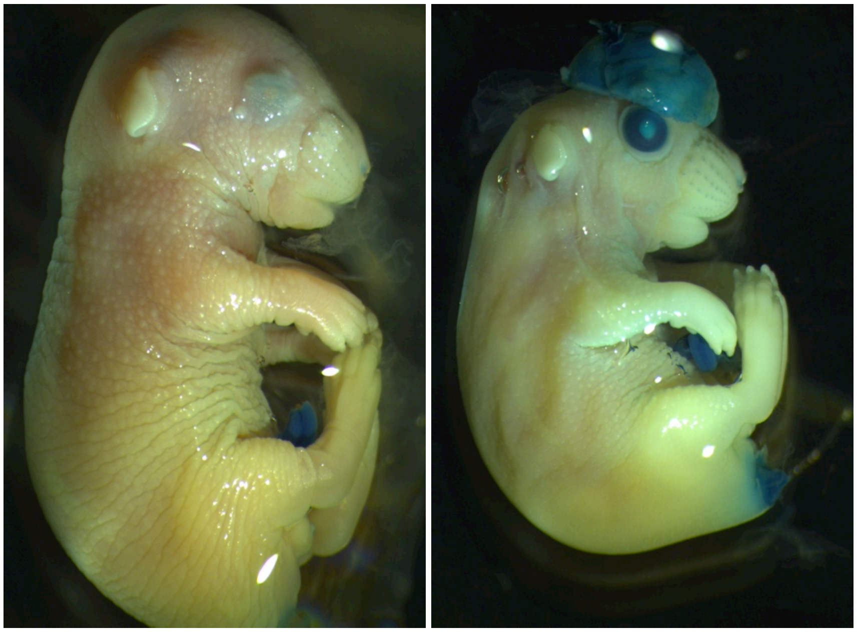

Figure S2.

To test the barrier function of *Colgalt1*$^{fosse/fosse}$ skin, we conducted a skin barrier assay (23). This test makes use of the intrinsic beta-galactosidase activity of the skin at its basal layers; normal skin will keep the X-gal out of the basal layers of the skin, while a disrupted skin barrier will allow the X-gal to reach these basal layers, where beta-galactosidase will catalyze the substrate to a blue by-product, turning the skin blue. The skin of wild type (left panel) and *Colgalt1*$^{fosse/fosse}$ (right panel) E18.5 embryos are not stained when treated with X-gal, demonstrating the skin barrier in the mutant is intact. As shown, mutant embryos demonstrate variable exencephaly and open eyes.
